# Protein Self-Assembly as a Design Principle for Enzyme Immobilisation in Biocatalysis

**DOI:** 10.3390/ijms27209187

**Published:** 2026-10-16

**Authors:** Santhosh Vijayakumar, Anwar Sunna

**Affiliations:** 1 School of Natural Sciences, Macquarie University, Sydney, NSW 2109, Australia; santhosh.vijayakumar@mq.edu.au; 2 Australian Research Council Industrial Transformation Training Centre for Facilitated Advancement of Australia’s Bioactives (FAAB), Sydney, NSW 2109, Australia

**Keywords:** enzyme immobilisation, protein self-assembly, biocatalysis, carrier-free immobilisation, catalytically active inclusion bodies, multi-enzyme cascades

## Abstract

Enzyme immobilisation is essential for translating biocatalytic processes from the laboratory to industrial applications by improving enzyme stability, recovery, and reuse. It underpins sustainable processes in pharmaceuticals, biofuels, and food production, but conventional immobilisation strategies involve inherent trade-offs. Covalent attachment prevents enzyme leaching but may distort the active site, whereas physical adsorption better preserves enzyme conformation but allows enzyme leaching. Furthermore, carrier-based methods frequently encounter mass-transfer limitations and may require external cross-linkers, hindering broader industrial application. Recent advances in synthetic biology and materials science have accelerated the development of protein-based materials for biocatalysis. This review examines protein self-assembly as a unifying design principle for enzyme immobilisation, enabling programmable assembly under relatively mild conditions to overcome key limitations of conventional strategies. We discuss naturally derived structures such as bacterial microcompartments and engineered systems including virus-like particles, elastin-like polypeptides, and SpyTag/SpyCatcher, which support programmable enzyme organisation and enable compartmentalised multi-enzyme cascade reactions. Building on this, a major focus is placed on catalytically active inclusion bodies (CatIBs). As robust, intrinsically self-immobilised protein aggregates, CatIBs represent a simplified, carrier-free platform offering potential process and cost advantages for industrial biocatalysis.

## 1. Enzyme Immobilisation in Modern Biocatalysis

Proteins are central molecular components that underpin numerous biological processes, both in vivo and in isolated systems, including cellular signalling and enzymatic catalysis. Owing to their unique physicochemical properties and functional diversity, proteins represent a promising class of sustainable biomaterials [1]. Among these, enzymes constitute a distinct class of proteins whose catalytic activity underpins both biological processes and a wide range of technological applications. For centuries, enzymes have served as an invaluable tool in the synthesis of high-value compounds. Biocatalysis has translated numerous enzyme-mediated reactions from laboratory studies to commercial processes, supporting applications in pharmaceuticals, fine chemicals, and food technology [2]. Enzyme immobilisation is a key strategy within biocatalysis that often involves multidisciplinary approaches to devise methods for the spatial confinement and functional integration of enzymes in a fixed space [3]. Free enzymes in solution, while catalytically efficient under optimal laboratory conditions, suffer from poor stability, difficult recovery, and single-use economics that render them impractical for most large-scale applications [4,5]. While conventional enzyme immobilisation has traditionally been viewed primarily as an operational tool designed to facilitate biocatalyst reuse, streamline product purification, and enable continuous operation in various reactor setups, this perspective has progressively shifted. Immobilisation is now increasingly recognised as a sophisticated engineering strategy used to enhance the biocatalyst itself [6,7,8]. Beyond confinement, immobilisation on appropriately functionalised supports can, in certain systems, enable the binding and partial purification of target enzymes directly from crude cell lysates [8]. Additionally, engineering the enzymatic nano-environment can modulate catalytic behaviour, including measurable gains in enantioselectivity for promiscuous enzymes without the need for extensive protein redesign [6]. However, immobilisation does not universally improve catalytic performance across all enzyme classes, as gains in stability and reusability can be offset by mass-transfer limitations, restricted substrate access, or immobilisation-induced conformational changes, which partly explains the variability reported across immobilisation studies [9,10]. These recurring limitations reflect a common underlying constraint, where immobilisation performance is governed by the interplay between enzyme accessibility, structural confinement, and diffusional transport, rather than immobilisation chemistry alone.

This variability highlights a critical need to develop fundamentally new design principles for enzyme immobilisation. In this context, immobilisation can transform relatively less stable, soluble enzymes into more robust, heterogeneous catalysts and help mitigate environmental and economic challenges inherent in industrial chemistry. From a technical standpoint, immobilisation has been reported to offer several practical advantages. First, immobilisation can enhance operational stability, shielding the enzyme’s structure from denaturation caused by extreme conditions such as elevated temperature, non-native pH, or exposure to organic solvents commonly encountered in industrial reactors, thereby extending its functional lifespan and half-life [10,11]. Second, it enables improved reusability; by confining the enzyme to an inert solid support (via adsorption, covalent bonding, entrapment, or cross-linking), the biocatalyst can be easily separated from the product mixture through simple filtration or centrifugation and reused for numerous production cycles, reducing the high cost associated with single-use enzymes [11,12,13]. Third, the spatial confinement facilitates streamlined downstream processing; the enzyme is removed cleanly from the reaction medium, minimising product contamination and substantially reducing the need for complex, energy-intensive purification steps [13,14]. Finally, the immobilised enzymes are well suited for continuous operation in advanced bioreactor designs, such as packed-bed or fluidised-bed reactors, allowing for improved process control, higher space-time yields, and easier integration into automated, large-scale industrial workflows [15].

Effective enzyme immobilisation techniques can be evaluated within the Safe and sustainable-by-design (SbD) principles of modern biotechnology. The transition of biocatalytic processes to large-scale commercial application, supported by the enhanced stability and reusability afforded by enzyme immobilisation, may contribute to sustainability objectives, including those related to industrial innovation, responsible production, and environmental impact [16]. From a Safe and sustainable-by-design perspective, these material- and chemistry-intensive strategies raise unresolved concerns related to lifecycle burden, leachable components, end-of-life handling, and regulatory acceptability, particularly for food, pharmaceutical, and environmental applications.

Enzyme immobilisation strategies are commonly classified according to the nature of the molecular interactions between the enzyme and the support material. These include non-covalent (relying on physical adsorption or physical confinement such as entrapment), covalent (involving strong chemical bond formation or surface bonding) or a hybrid approach utilising both mechanisms. Each mechanism presents distinct trade-offs between ease of immobilisation, enzyme-support bond strength, activity retention, and stability improvement. Despite the advantages, traditional immobilisation approaches relying on chemical cross-linkers or carriers often introduce drawbacks that may hinder scalability. Carrier-based methods involve attaching enzymes to inert supports like inorganic, organic, nanoscale, polymeric, or ceramic platforms via adsorption, entrapment, or covalent bonding. Table 1 summarises the carrier classes and their key properties that influence immobilisation.

Conceptually, these approaches may be limited by mass transfer restrictions depending on carrier structure and operating conditions, where substrate diffusion to the enzyme active site may be impeded by carrier porosity and internal architecture, leading to suboptimal kinetics. Additionally, carrier dilution reduces volumetric activity, as a significant portion of the system is non-catalytic material, and scalability is challenged by the need for uniform loading without aggregation. Importantly, among these constraints, mass transfer limitations and loss of orientational control represent key design bottlenecks that directly impact catalytic efficiency and space–time yield, whereas cost, toxicity, and regulatory challenges depend on carrier composition, cross-linker chemistry, and the intended application domain (e.g., food, pharmaceutical, or environmental use).

The recent comprehensive review by Holyavka and Artyukhov [17] systematically analyses the conceptual limitations of carrier-based immobilisation and identifies key disadvantages and areas for future methodological development. These methods can lead to reduced enzyme activity through conformational distortions or active-site blockage, are in some systems associated with enzyme leaching, escalate costs due to expensive materials and complex procedures, and may pose toxicity risks arising from residual cross-linkers or leached carrier components, particularly in food or pharmaceutical applications [17,18]. Overall, these limitations arise from the reliance on extrinsic carrier materials and chemical linkers, which externalise control over enzyme organisation and impose structural, diffusional, and chemical heterogeneity that are not always fully resolved through incremental optimisation of immobilisation chemistry alone.

Cross-linker-mediated covalent attachment of enzymes onto solid surfaces commonly utilises amide bond formation via carbodiimide chemistry or imine (Schiff-base) linkages. Carbodiimide-mediated coupling enables amide bond formation between carboxyl groups and primary amines present on either the enzyme or the carrier surface. Despite the superior strength and stability of these covalent linkages, carbodiimide-mediated immobilisation can, in certain cases, lead to unintended self-cross-linking from reactive carboxylic acid and amine groups within enzyme molecules. Moreover, the carbodiimide reaction proceeds through a two-step activation process, in which a typical carbodiimide reagent, EDC (1-ethyl-3-(3-dimethylaminopropyl) carbodiimide hydrochloride), in combination with NHS (N-hydroxysulfosuccinimide), activates the support’s carboxylic acid to form an O-acylisourea intermediate, followed by coupling of this intermediate with the enzyme’s primary amine group, releasing a water-soluble isourea byproduct whose toxicity is context-dependent [19]. Glutaraldehyde and related bifunctional reagents that mediate Schiff base reactions have been employed as cross-linkers in many enzyme immobilisation studies. These reagents provide strong enzyme-support interactions but are associated with several reported limitations, including reversibility of the bonds, potentially irreversible modification of enzyme residues that can distort active sites and diminish specificity, variable cross-linking efficiency, conformational restriction, and creation of diffusion barriers within densely crosslinked networks [20,21]. These methods also lack tunability for multi-enzyme systems, often resulting in limited control over enzyme orientation that can impair active-site accessibility, and they introduce chemical heterogeneity that can complicate downstream purification and regulatory assessment. These limitations have motivated the development of alternative, more sustainable cross-linking strategies. While several new options have been introduced, a universal cross-linker that demonstrates compatibility across a wide range of enzymes and supports remains an ongoing research opportunity [22]. These challenges suggest that further progress in biocatalyst engineering may increasingly benefit from complementary design approaches beyond purely carrier- and cross-linker-centric paradigms toward approaches that encode structural organisation, stability, and functionality directly within the protein system itself.

Several recent reviews have examined enzyme immobilisation strategies, particularly comprehensive overviews of carrier-based systems and their associated limitations [17,23], with a primary focus on externally added solid supports and cross-linking chemistries. Recent reviews have also examined protein self-assembly in artificial organelles, including protein cages, scaffolds, and membraneless condensates [24], and the rational spatial assembly of multi-enzyme cascades across biological and non-biological platforms [25]. The former primarily emphasises assembly mechanisms, compartmentalisation, and metabolic organisation, whereas the latter focuses principally on spatial organisation and cascade performance across diverse carrier classes. More focused reviews have addressed modular systems such as SpyTag/SpyCatcher [26]. Inclusion bodies and catalytically active inclusion bodies (CatIBs) have also been reviewed as functional biomaterials and carrier-free self-immobilising systems, primarily in the context of protein aggregation and recombinant expression platforms [27,28,29]. However, these areas have generally been treated independently, without a unified framework that connects immobilisation principles with biologically encoded self-assembly strategies. In contrast to these existing reviews, which treat immobilisation strategies, self-assembling protein systems, and inclusion bodies as largely separate domains, this review explicitly integrates these areas by positioning protein self-assembly as a unifying framework for enzyme immobilisation in biocatalysis, with particular emphasis on architecture–function relationships and the role of CatIBs within this broader design landscape. By doing so, it enables a more unified interpretation of structure–function relationships, spatial organisation, catalytic microenvironment, and process-relevant performance across these systems.

**Table 1:** Advantages, limitations, and applications of common enzyme carriers.

Carrier Type	Material	Key Properties	Pros and Cons	Applications	References
Porous inorganic carriers	Controlled pore glass, silica, ceramic, metal oxides	High mechanical strength, large surface area, stable (pH/temperature); long shelf life	Regenerable; limited by mass transfer constraints and potential enzyme leakage	Membrane reactors, immobilised enzyme membrane modules	[10]
Nanoparticles/magnetic particles	Iron oxide magnetic nanoparticles, magnetic CLEAs, carbon nanotubes, metal nanoparticles	High surface-to-volume ratio	Magnetic separation enables speed and convenience; risks include leaching, aggregation, and potential activity loss during surface modification	Bioconversion requiring rapid solid–liquid separation	[5,30]
Polymeric carriers (synthetic and renewable)/hydrogels	Polyethyleneimine coatings, alginate beads, chitosan, cellulose, polymethacrylate, polystyrene-divinylbenzene	Tuneable porosity and surface chemistry, variable mechanical properties	Biocompatible, low cost, allows covalent attachment; generally lower mechanical strength	Synthetic resins are used industrially for the immobilisation of lipases, proteases, and other enzymes in food and detergent applications	[31]
Metal–organic frameworks (MOFs)	Enzyme and MOF composites	Ordered porous structure, high surface area, tuneable pore size	Can mitigate mass transfer resistance depending on pore architecture; some MOFs are limited by stability in aqueous media and high industrial cost	Emerging applications in fine-chemical catalysis and tailored flow reactors	[32]

## 2. Protein Self-Assembly as a Design Principle

In nature, many proteins exhibit an inherent ability to self-assemble into complex, higher-order structures that play critical roles in cellular organisation, structural integrity, and functional compartmentalisation. This process is driven by non-covalent interactions, including hydrogen bonding, electrostatic forces, and hydrophobic effects, and proceeds under thermodynamic control to yield structurally defined supramolecular architectures [33]. Representative examples include protein components that form supramolecular assemblies within cells, such as cytoskeletal elements, viral capsids, and bacterial microcompartments [34]. In the context of biocatalysis, this natural propensity has been harnessed to develop protein-based scaffolds for enzyme immobilisation. Protein-based platforms that leverage self-assembly provide biocompatible, site-specific scaffolds in which catalytic performance is strongly influenced by architecture-dependent control of enzyme localisation, stoichiometry, and microenvironment, and in some systems can enhance stability and improve catalytic efficiency. Key advantages include the potential for reduced mass-transfer limitations via tuneable pores or open structures, stability enhancement mediated by protective intermolecular interactions (e.g., hydrogen bonding or hydrophobic shielding) in systems where confinement or packing does not compromise substrate access to the active site, and ease of preparation via genetic fusion or spontaneous assembly, thereby reducing reliance on complex chemical immobilisation strategies. Importantly, the extent to which self-assembly enhances stability or catalytic efficiency is architecture-dependent, with scaffold porosity, enzyme packing density, and spatial organisation determining whether confinement stabilises the enzyme or introduces new diffusional or steric constraints [35,36]. Across these systems, protein self-assembly shifts enzyme immobilisation from an externally imposed constraint to an encoded architectural feature, enabling control over enzyme localisation, stoichiometry, and microenvironment that can be difficult to achieve using carrier-based or chemically cross-linked approaches [37,38,39].

**Figure 1:**
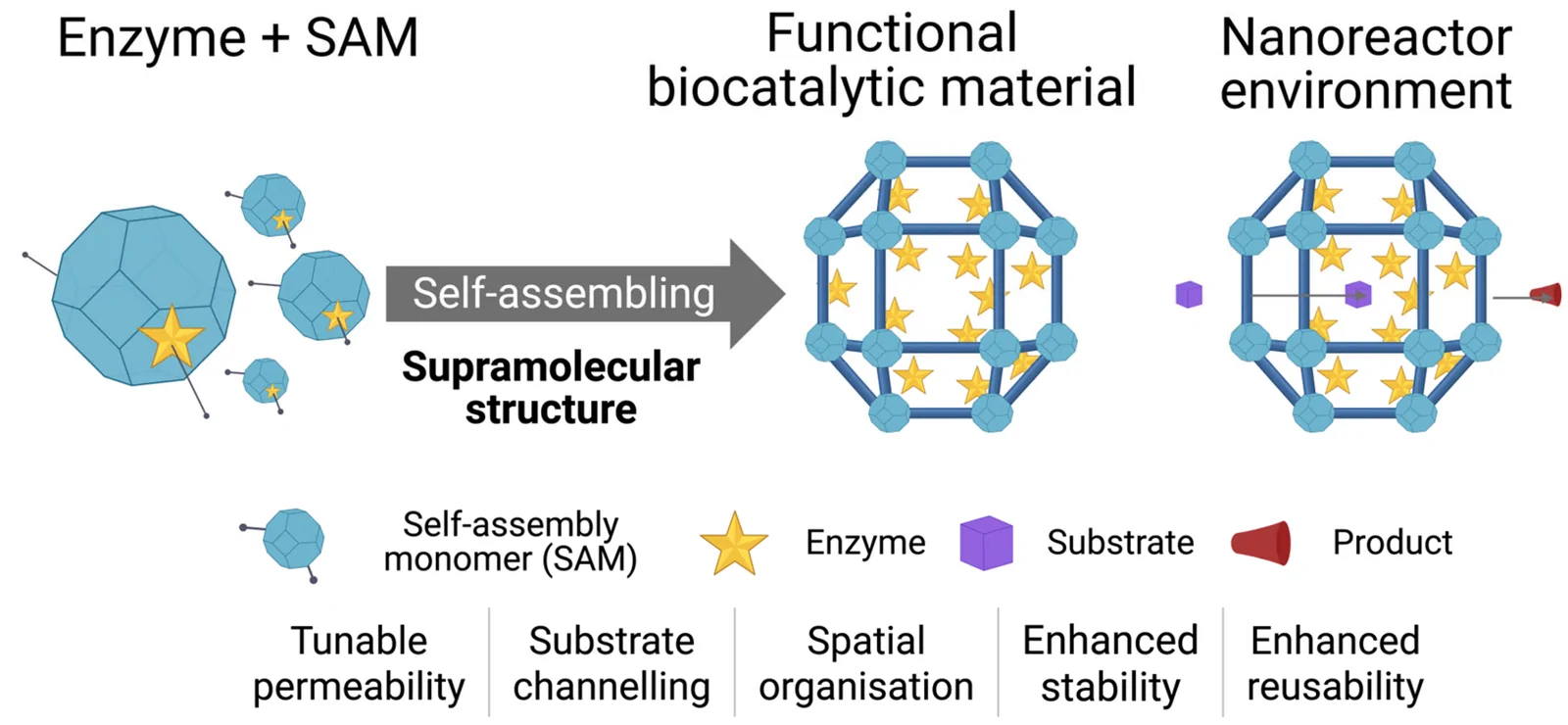
Illustration of the basic protein self-assembly process for enzyme immobilisation and its advantages. Created in BioRender. Sunna, A. (2026) https://BioRender.com/5dm3srh.

**Figure 2:**
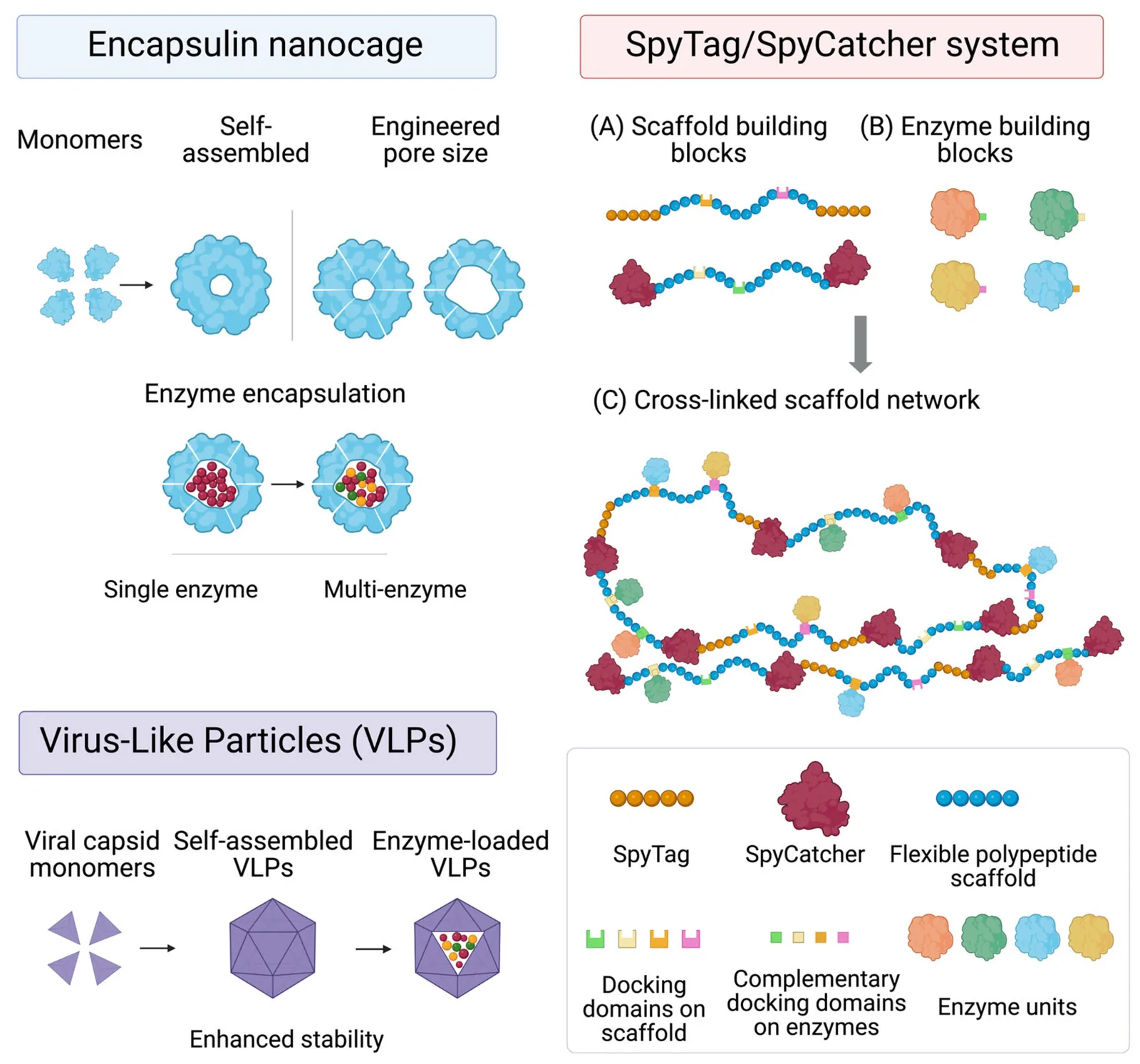
Illustration of different self-assembly systems for enzyme immobilisation. Created in BioRender. Sunna, A. (2026) https://BioRender.com/wz839ue.

The use of biological self-assembly as a complement to chemical cross-linking reflects the ability of biomolecular systems to encode structural organisation through sequence and assembly design. Protein self-assembly process can be genetically encoded, while protein scaffolds can be combined with biomolecular conjugation strategies, including nucleic-acid-mediated cargo organisation, allowing controlled enzyme localisation and defined stoichiometry within the scaffold (Figure 1). This programmability enables the co-localisation of multiple enzymes with precise spatial separation in multi-enzyme cascades, thereby in some systems reducing intermediate diffusion [40,41,42]. Many protein self-assembly systems can be formed under relatively mild, aqueous conditions, avoiding the harsher conditions required for some chemical immobilisation strategies. Such conditions can help preserve native enzyme structure and catalytic activity. The resulting tuneable protein assemblies function as stable nanoreactors, which in certain architectures can reduce substrate inhibition by modulating local substrate concentrations. This in vivo or in vitro assembly approach facilitates site-specific enzyme attachment, which in appropriately designed systems can promote substrate channelling in multi-enzyme cascades and can enhance overall reaction kinetics [35,41,43]. The generic biocatalytic advantages and key properties of various self-assembling systems are summarised in Table 2, while Figure 2 illustrates representative protein self-assembling platforms for enzyme immobilisation. The following examples illustrate how protein-based immobilisation can address several longstanding limitations of conventional immobilised enzymes, including stability, mass transfer, and spatial organisation in multi-enzyme cascades.

The fusion system comprising collagen-like polypeptide (CLP) and elastin-like polypeptide (ELP) was used to construct a proteinaceous microporous network (PMN) of superoxide dismutase (SOD) and D-amino acid oxidase (DAAO). The PMN-based catalysis demonstrated reduced by-product (H_2_O_2_)-induced oxidative deactivation, which was associated with an approximately 1.7-fold increase in the catalytic efficiency of DAAO. The confined enzymes within the PMN also showed improved stability against urea denaturation and excellent reusability, retaining ~80% activity after 10 cycles, exceeding the performance of the corresponding free enzymes [44]. A self-assembly strategy was employed to construct multi-enzyme nanostructures on ELP protein scaffolds that act as flexible backbones integrated with the SpyTag/SpyCatcher (ST/SC) system for covalent cross-linking and cyclisation. High enzyme density was achieved through ST/SC-mediated assembly, while orthogonal docking domains incorporated into the ELP backbone enabled modular attachment of menaquinone pathway enzymes (MenF, D3’-MenD, D4’-MenH). These architectures achieved product yields up to 94% compared to free enzymes, which was attributed to architecture-dependent flux streamlining (lower *k*_m_) in cross-linked assemblies and enhanced catalytic efficiency (higher *k*_cat_) in cyclic scaffolds, demonstrating architecture-dependent control over biocatalytic performance [45].

Pore engineering of self-assembled bacterial encapsulin nanocompartments enabled an expanded permeability window for larger substrates and cofactors, improving molecular flux and mitigating pore-substrate/cofactor size mismatch. In pore-engineered Myxococcus xanthus encapsulin nanoreactors, encapsulated alcohol dehydrogenase D (AdhD) exhibited improved performance relative to wild-type shells, while co-encapsulation of the cofactor-recycling enzyme phosphite dehydrogenase (PTDH) resulted in an approximately 84% increase in turnover in the engineered shells. Notably, free AdhD retained higher intrinsic activity than the encapsulated enzyme, highlighting a common trade-off in nanoreactor systems whereby pore engineering alleviates, but does not fully eliminate, confinement-associated activity penalties [39]. Another multi-enzyme platform based on engineered virus-like particles (VLPs) enabled co-encapsulation of a dehydrogenase with a partner reductive enzyme system, resulting in efficient coupled reactions for multi-step reduction chemistry. The high enzyme packing density and colocalization within the VLP nanoreactor increases effective proximity, which in appropriately designed systems can contribute to increased overall reaction rates via substrate channelling [38]. The genetic platforms used in both systems allowed relatively straightforward increases in encapsulated enzyme payload, which can improve overall biocatalytic efficiency and volumetric productivity [38,39]. Across these examples, self-assembled protein scaffolds have been shown to improve catalytic efficiency, operational stability, and reusability relative to the corresponding soluble enzymes, with the extent and mechanism of enhancement dependent on scaffold architecture and enzyme identity [38,39,44].

In bacteriophage P22 VLPs that can adopt procapsid (PC) and expanded (EX) morphologies, effective pore sizes of 4.4 nm (PC) and 2.7 nm (EX) have been reported [36], enabling controlled molecular access and size/charge-dependent partitioning. Substrate selectivity arises from Coulombic partitioning within the negatively charged framework microenvironment, favouring positively charged molecules while excluding negatively charged species, and this partitioning can be tuned by ionic strength.

Immobilisation using low-toxicity amyloid-like protein assemblies has been demonstrated through both direct self-assembly and hydrogel formation. For example, the ability of a prion domain, Ure2, to self-assemble into fibrillar aggregates has been utilised to co-immobilise alkaline phosphatase and horseradish peroxidase. The resulting chimeric fibrils were applied onto an agarose column for repetitive flow catalysis [46]. In a hybrid self-assembly/cross-linking approach, β-lactoglobulin, which forms amyloid fibrils under acidic conditions, was cross-linked using dimethyl pimelimidate dihydrochloride (DMP) to generate a hydrogel. This hydrogel immobilised a seven-enzyme system constituting an artificial, photosynthesis-inspired multi-enzyme pathway for fructose synthesis from sequestered CO_2_, with sustained activity and long-term stability [47]. Similarly, self-assembled Q11 peptide nanofibres have been developed as artificial catalysts for ester hydrolysis [48], while Fmoc-Y-OMe-based hydrogels have been used to immobilise enzymes, including PETase, resulting in enhanced polyethylene terephthalate degradation at 70 °C [49]. A recurring observation in peptide-, protein-, and cage-based self-assembled scaffolds is an observed expansion of the functional stability window relative to free enzymes, particularly under extremes of temperature, pH, or chemical stress, underscoring the role of confinement and scaffold-mediated interactions in preserving enzymatic structure and activity [44,47,49].

Despite these advantages, protein self-assembly does not universally guarantee improved biocatalytic performance, as catalytic outcomes are constrained by recurring trade-offs between diffusion, enzyme packing density, and scaffold architecture across different systems. Excessive confinement, overly dense packing, or unfavourable pore architectures can restrict substrate access, hinder product release, or impose conformational strain that compromises activity. Several studies highlight that beneficial effects emerge only within defined architectural windows, where scaffold porosity, enzyme loading density, and interaction strength are balanced to preserve catalytic accessibility while providing stabilisation [35,36,39].

Due to their self-assembly properties, hydrophobins, which form stable protein films at interfaces, and self-assembling amino acid hydrogelators that form bulk nanofibrous networks, represent promising systems for enzyme immobilisation. Despite their potential for controlled enzyme immobilisation, biocatalytic applications of hydrophobins remain underexplored [50]. Self-assembling hydrogels derived from Fmoc-protected phosphorylated tyrosine methyl ester (Fmoc-pY-OMe) enable enzyme-instructed self-assembly via non-covalent interaction within nanofibrous networks. The hydrogelator-based immobilisation achieved high encapsulation efficiency (~88–91%) with enzymes such as lipase, alkaline phosphatase (ALP), leucine aminopeptidase (LAP), β-galactosidase (β-Gal), and PETase. This nanofibrous network can provide protection against harsh conditions, with the hydrogels remaining stable across pH 1–9 and at temperatures up to 70 °C while preserving enzymatic activity. For instance, immobilised β-Gal retains 63% activity at pH 1 (vs. 42% for free β-Gal), and PETase degrades polyethylene terephthalate (PET) film at 70 °C, a temperature matching PET’s glass transition point, while free PETase is inactive [49].

Cross-β-sheet rich fibrils formed by silk fibroin have been used as a biocompatible matrix for entrapment of various enzyme classes, including oxidoreductases, transferases, hydrolases, and lyases. A detailed review by Lv [51] summarised multiple silk-fibroin-based immobilisation strategies for different enzymes, which in many reported systems conferred enhanced stability and reusability, with improvements in catalytic efficiency observed in specific systems. These examples illustrate the versatility of protein-based materials in biocatalytic applications.

SpyTag/SpyCatcher (ST/SC) is a modular protein assembly system mediated by spontaneous covalent isopeptide bond formation between an aspartate residue in SpyTag and a lysine residue in SpyCatcher. While existing reviews have described cases in which ST/SC systems outperform free enzymes in selected applications [26], the following discussion focuses on how this platform is applied in multi-enzyme cascades, with emphasis on key process metrics. In multi-enzyme cascades, ST/SC can contribute to flux optimisation by enabling directed co-assembly of sequential enzymes through genetic fusion to tags or scaffolds, which is consistent with reduced intermediate diffusion distances. For instance, maltooligosyl trehalose synthase (MTS) and maltooligosyl trehalose trehalohydrolase (MTH) were assembled in vitro into a supramolecular complex, accelerating trehalose production from maltodextrin with a 58.5% conversion rate in 40 h, peaking 10 h earlier than free enzymes [52]. More recently, a self-assembling γPFD-SpyCatcher hydrogel provided a protein scaffold for co-immobilisation of SpyTag-fused enzymes, with a maximum loading ratio of 1:1 and improved thermal and pH stability. In a cell-free system, co-immobilisation increased pyruvate production by 43–78%, depending on the immobilised enzyme, while co-immobilised glutamate dehydrogenase displayed 38% higher activity than the purified enzyme [53]. A subsequent in vitro ST/SC-assembled dextransucrase–dextranase cascade achieved 97.7% sucrose conversion, a 41.7% higher reaction rate than the free-enzyme mixture, and an estimated intermediate-transfer efficiency of 62.2% compared with 2.6% for the free system [54].

Across protein cages, VLPs, peptide hydrogels, amyloid fibrils, and modular covalent assemblies described in this section, biocatalytic performance is often governed by mesoscale architectural features that mediate the balance between substrate diffusion, enzyme packing density, and confinement effects, rather than immobilisation chemistry alone. Despite their diversity, these self-assembling platforms often retain architecture-dependent trade-offs, including residual diffusion constraints, confinement-associated activity penalties, and reliance on extrinsic scaffolds, hybrid materials, or multicomponent assembly strategies that complicate scalability, manufacturability, and application-specific deployment. These recurring limitations motivate examination of carrier-free protein materials in which immobilisation, structural organisation, and catalytic function are encoded directly within the enzyme construct itself, providing a rationale for considering CatIBs as a distinct class of self-assembled biocatalytic materials.

**Table 2:** Natural and engineered self-assembling protein and peptide scaffolds for biocatalysis.

Protein	Structural Class	Self-Assembly Mechanism	Key Advantages	Biocatalytic Application	References
Silk fibroin	Structural protein	β-sheet-rich assembly via hydrogen bonding and hydrophobic interactions	Biocompatible, high loading capacity, high enzyme retention can be achieved	Enzyme immobilisation in biosensors and recyclable biocatalysts	[51,55]
Collagen and Collagen-like Peptides	Extracellular matrix	Self-assembles into triple-helical fibers via electrostatic and hydrophobic forces	Improves thermal stability, reusability, and reduces enzyme degradation	Immobilisation into films/hydrogel	[44,56,57]
Shell protein scaffold	Bacterial microcompartment	Hexameric or pentameric units assemble into cages via protein–protein interfaces	Modular, genetic tunability, improves cascade efficiency; stability; easy separation	Co-immobilisation providing suitable pH microenvironment, improved stability	[58,59]
Encapsulin	Self-assembling protein nanocage	Subunits self-assemble into icosahedral shells via protein–protein interactions	Protects enzymes from denaturation and proteolysis, improves catalytic activity and substrate flux via engineered pores; programmable enzyme packaging	Nanoreactors for encapsulating enzymes in multi-enzyme cascades, enabling cofactor recycling (e.g., NADH)	[39,60,61]
Virus-like particles	Self-assembling protein nanocage	self-assemble into capsid-like protein structures via protein–protein interactions during heterologous expression	Nanoreactors for encapsulating enzymes	Enzyme encapsulation (e.g., cytosine deaminase, peptidase E, and purine nucleoside phosphorylase); substrate channelling	[37,38]
Hydrophobins	Amphiphilic extracellular proteins	Interfacial self-assembly into amphipathic mono-layers at hydrophobic–hydrophilic interfaces	Modular; high surface activity; structural hierarchy	Surface enzyme immobilisation and biosensing (e.g., caffeic acid detection)	[50,62,63]
Amyloid fibrils	Self-assembling protein fibrils	Self-assembly into cross-β amyloid fibrils; fibrils can further form hydrogel networks	High stability; easy recovery; reusability	Reusable enzyme-displaying fibrils and hybrid amyloid hydrogels for multienzyme cascade biocatalysis	[46,47]
Elastin-like polypeptide (ELP)	Thermo-responsive biopolymer	Temperature-responsive coacervation via hydrophobic collapse above transition temperature	Biocompatible, genetically programmable, enhances solubility and stability	Forms self-assembling scaffolds for multienzyme cascades, with tuneable phase behavior	[33,44,64]
SpyTag/SpyCatcher system	Covalent self-assembly system	Covalent isopeptide bond formation between the SpyTag peptide and SpyCatcher protein domain, enabling modular assembly	Site-specific; rapid assembly under mild conditions; high fidelity	Co-immobilisation of cascade enzymes on scaffolds; high-activity biocatalysis	[26,45,52,53,65]

## 3. Inclusion Bodies as a Natural Example of Protein-Based Biomaterials

Heterologous gene expression in *Escherichia coli* is a robust platform for producing recombinant proteins and enzymes in laboratory and industrial settings. However, a major challenge has historically been the formation of insoluble inclusion bodies (IBs), dense protein aggregates long regarded as non-functional biological waste. Recent research has challenged this view, showing that IBs can contain substantial amounts of active, native-like folded protein species. While initial efforts focused on extracting soluble protein from IBs, there is increasing interest in utilising certain IB preparations directly. Their favourable biophysical properties, including sub-micrometer particle size and the ability to act as carrier-free protein immobilisates, make IBs viable biomaterials and potential nanocatalysts, with scope to reduce drawbacks associated with traditional enzyme immobilisation methods. Because CatIBs combine intracellular formation, carrier-free immobilisation, and simplified recovery, they are examined in greater detail here as a distinct platform within the broader spectrum of protein self-assembly strategies.

As discussed, traditional immobilisation of soluble enzymes using carriers or cross-linkers often requires case-by-case optimisation and can be cost-intensive [9,14]. In this context, CatIBs represent a subclass within protein-based immobilisation in which IBs function as biologically produced, carrier-free immobilisates. CatIBs can form during heterologous overexpression in *E. coli*, thereby integrating enzyme production and immobilisation into a single process step. This can reduce the need for chromatographic purification and/or additional immobilisation steps in some workflows [27]. However, reported CatIB residual activity can vary substantially depending on the aggregation-inducing fusion partner and resulting packing density, spanning from low single-digit percentages to approximately 10–18% of the corresponding soluble enzyme in systematic comparisons of different fusion designs [66]. While CatIBs possess useful attributes as a carrier-free immobilisate, the production of highly active CatIBs remains dependent on the selection of appropriate aggregation-inducing fusion partners, as discussed below.

The conceptual benefit of CatIBs lies in their ability to occupy an intermediate position between low-cost carrier-based immobilisation and more structurally sophisticated protein self-assembly systems. Table 3 contrasts the operational benefits and trade-offs of CatIBs with those of other self-assembly systems, highlighting their stability and simplified downstream processes.

1. Reduction in the purification bottleneck: Downstream purification represents a major cost and complexity contributor in enzyme technology. CatIBs can substantially reduce this burden by enabling the direct use of aggregated, catalytically active protein following cell lysis and minimal processing, rather than requiring full chromatographic purification. This feature simplifies immobilisation workflows and may improve overall process efficiency and streamline processing requirements. In this context, CatIB production was explicitly contrasted with cross-linked enzyme aggregates (CLEA)-type workflows, as CatIBs required only cell lysis, centrifugation, and washing steps, whereas CLEA preparation involved enzyme purification, precipitation, and chemical cross-linking [66].
2. In vivo formation of robust biocatalysts: Unlike some engineered self-assembly systems that require post-expression purification and in vitro enzyme loading [67], CatIBs form their solid, mechanically stable structure intracellularly during heterologous expression. The resulting aggregates can be recovered after cell disruption as carrier-free immobilisates with minimal processing, integrating enzyme production and immobilisation into a single biomanufacturing step [27,66].
3. High-capacity co-immobilisation for enzyme cascades: While engineered cages provide precise stoichiometric control, CatIBs enable high-capacity, albeit random, co-immobilisation through co-expression of multiple enzymes. Such mixed aggregates have been shown to support multi-enzyme cascade reactions and can enhance overall cascade efficiency, reflecting reduced diffusion distances arising from high local enzyme concentrations, making CatIBs a practical and scalable option for complex biocatalytic pathways [68].

**Table 3:** Comparison of CatIBs with other engineered self-assembly systems.

Feature	CatIBs	Other Engineered Self-Assembly Systems	References
Preparation/downstream processing	Expression-driven formation; recovered by cell lysis, centrifugation and washing; chromatographic purification generally avoided	System-dependent; may require scaffold purification and/or in vitro assembly or loading, although some systems assemble in vivo	[27,39,44,66,67,69]
Loading/activity retention	Carrier-free; often high protein content; residual activity strongly construct-dependent (~1–18% in a systematic fusion-design comparison)	High cargo loading can be achieved, but confinement and accessibility may reduce activity (e.g., 70–98% loading; free enzymes ~2–3-fold more active in an encapsulin system)	[39,66,68,69]
Stability	Enhanced stability can be achieved, but is enzyme- and construct-dependent	Stability enhancement varies with scaffold architecture and enzyme	[27,39,44,67,68,69,70]
Reusability	Repeated use demonstrated; recovery by centrifugation or, after magnetisation, magnetic separation (up to 50 cycles)	Repeated use demonstrated; recovery depends on scaffold format	[27,44,46,69,71]

## 4. Functional Inclusion Bodies: Current Knowledge, Engineering Principles, and Remaining Challenges

Contrary to early views of IBs as inactive aggregates, multiple studies have reported the presence of structured microdomains and native-like folding cores within IBs, supporting partial and, in some systems, retained functional or catalytic activity. The precise mechanism of IB formation remains debated, yet current evidence suggests it is protein-specific and initiated by the misfolding of kinetic folding intermediates [72,73]. Furthermore, in vivo studies reveal a dynamic equilibrium within the host’s protein homeostasis, driving simultaneous IB aggregation and disintegration [74,75]. This dynamic behaviour supports the view of IBs as functional intermediates rather than inert byproducts in recombinant protein production [76]. Activity retention is influenced by expression conditions, fusion design, and internal IB architecture. Early investigations demonstrated that culture conditions, including high temperature, induction timing, salt concentration, and aeration, strongly influence the formation of enzymatically active β-galactosidase IBs [77,78]. The production of human granulocyte-colony stimulating factor (hG-CSF) in *E. coli* at lower temperature resulted in a distinct “non-classical” type of IB containing substantial amounts of correctly folded protein, from which biologically active hG-CSF could be extracted under mild, non-denaturing conditions [79]. Subsequent studies demonstrated that tuneable IBs can be produced by systematically modifying cultivation parameters, including inducer concentration, cultivation pH, temperature, and induction time. Therefore, the intracellular microenvironment in which protein folding occurs appears to be an important determinant in IB formation. These observations provide a basis for exploring IBs as functional biomaterials [80,81,82,83].

### 4.1. Mechanistic and Structural Basis of Functional IB Formation

The study by Carrió et al. [84] first demonstrated the ordered organisation of β-galactosidase IBs (VP1LAC) formed by fusion to the VP1 capsid domain of foot-and-mouth disease virus (FMDV), which acts as an aggregation module. Their work reported a linear correlation between the amount of insoluble VP1LAC and average IB volume as a function of induction time, as well as the coexistence of large, growing IBs with newly appearing aggregates, consistent with mutually exclusive aggregation cores. This study primarily established the organisational and growth characteristics of VP1LAC IBs, providing a basis for subsequent engineered-IB approaches.

At the molecular level, research has elucidated important roles of hydrophobic collapse, β-sheet stacking, and aggregation hotspots in driving IB formation. Spectroscopic and microscopic analyses demonstrate amyloid-like cross-β structures coexisting with native-like domains within IBs, consistent with aggregation arising predominantly from partially folded intermediates rather than fully native or completely unfolded proteins [28,85,86,87]. The identification of molecular crowding effects and partially folded intermediates refines the mechanistic understanding of IB formation and provides a basis for rational strategies aimed at improving activity retention within IBs [80,88].

In recent years, computational tools and sequence-based analyses have enabled the identification of aggregation-prone regions (APRs) that contribute to IB formation, balancing evolutionary minimisation of aggregation with structural and functional constraints. Aggregation propensity correlates with protein stability, folding intermediates, and in some cases with proximity to catalytic residues, facilitating rational design and solubility optimisation. Predictive algorithms are therefore used to understand and engineer aggregation behaviour for biotechnological applications [89,90,91]. Such approaches have been used to identify short sequence segments that disproportionately dominate aggregation behaviour, enabling targeted mutagenesis or fusion design to modulate IB formation and functional outcomes [89,90].

High-resolution NMR and complementary FTIR analyses reveal extensive protection and substantial native-like structural features within IBs [92]. However, the extent of activity retention varies widely depending on protein type, expression conditions, and IB morphology, with some IBs exhibiting substantial conformational heterogeneity and reduced functionality. Reported activity retention spans a broad range, from near-complete loss to substantial fractions of the corresponding soluble enzyme, reflecting differences in aggregation pathways and structural organisation [80,82]. The mechanisms underlying activity loss in certain IBs are not fully understood, and the balance between stability and flexibility within IBs remains difficult to control. Additionally, the contribution of surface-adsorbed versus embedded active proteins to the overall catalytic output of IBs remains debated [85]. The observation that active protein forms within the core of IBs suggests that functional species can be integral components of the aggregate rather than passive contaminants, while the presence of functional species at the solvent interface is consistent with a porous architecture that facilitates environmental interaction despite protein aggregation. This structural heterogeneity complicates both the characterisation of IBs and the prediction of functional performance across different systems [93].

These mechanistic and structural features provide the basis for the engineering strategies discussed in Section 4.2.

### 4.2. Engineering Strategies for Functional IBs

Engineering strategies based on aggregation-inducing fusion tags and spacer linker length and flexibility have been explored to optimise functional IB formation and enzymatic activity in CatIBs. One approach to the modular construction of CatIBs involves separating the target enzyme from the aggregation-inducing partner using a spacer linker. This architecture can reduce steric constraints during folding and can improve the functionality of the resulting IBs, although these effects depend on linker length and flexibility. The strategies described below show improvements in activity and, in some cases, stability and reusability of IBs, with linker properties critically influencing aggregation efficiency and catalytic performance.

Building on these mechanistic insights, multiple studies demonstrated the formation of enzymatically active IBs generated via fusion to aggregation-inducing partners, including cellulose-binding domains [94], coiled-coil domains [69], and fluorescent protein-based aggregation motifs [95,96]. Additional studies have reported enzymatically active IBs across a wide range of enzymes and construct designs, supporting the applicability of fusion-driven IB formation beyond any single aggregation strategy [95,97,98,99,100,101,102,103]. Overall, this body of work indicates that fusion to aggregation-inducing partners can be an effective strategy for generating enzymatically active IBs, although activity retention varies with fusion architecture and expression conditions. This motivates the empirical identification and optimisation of aggregation-inducing partners and construct designs to obtain highly active CatIBs.

Diener and colleagues introduced the term “CatIBs” in 2016 following their method for producing active enzyme IBs. They utilised the parallel coiled-coil domain (TdoT) as a fusion partner to support CatIB formation for three structurally distinct enzymes. These resulting CatIBs exhibited favourable biocatalytic properties, demonstrating stability and applicability in both aqueous and organic reaction systems [69].

As discussed, the formation of CatIBs via heterologous expression in bacterial systems is dependent on the conditions applied. Specifically, the design of the fusion protein is a key determinant of residual activity, with systematic comparisons reporting ~1–2% versus ~10–18% residual activity for different fusion designs [66], and construct/process choices can also influence practical recovery and use [66,104].

Various natural and synthetic functional peptides, including surfactant-like and amphipathic self-assembly peptides, have been used as aggregation-inducing tags for IB production, supporting high expression levels and retention of catalytic activity, with stability or reusability improvements reported for specific designs [102,105,106]. In a limited number of cases, these short peptide tags have been combined with self-cleaving inteins or protease-cleavable SUMO tags, enabling controlled release of soluble protein from aggregated constructs and facilitating protein extraction [107,108,109].

In a study by Küsters and colleagues [106], the use of hydrophobic tags and rigid PT-linkers influenced CatIB morphology, resulting in enhanced activity across variants, high volumetric productivity, and improved stability for reusable biocatalysts, with linker choice enabling early strain screening. Similarly, Jäger et al. [66] evaluated linker deletions, fusion orientations, and tag variants, correlating hydrophobic surface area with aggregation behaviour and morphology, enabling more predictable CatIB formation through structure-based design.

Recent studies examined the impact of various functional peptides (ELK16, DKL6, L6KD, ELP10, ELP20, L6K2, EAK16, 18A, and GFIL16) of differing lengths when terminally fused to tyrosine phenol-lyase (TPL) in *E. coli*. The findings indicated that the length and type of these peptides significantly influenced the enzyme’s properties and overall performance [110]. By systematically optimising tag selection and fusion strategies, Ölçücü et al. [70] demonstrated the production of alcohol dehydrogenase CatIBs that exhibited superior activity and stability. Their work showed that the optimal combination of fusion site and aggregation tag is enzyme-specific, indicating that empirical optimisation remains necessary to maximise residual activity in CatIBs. Similarly, Tu et al. [111] investigated several self-assembling peptide fusions (ELK16, L6KD and 18A) to generate functional fluorinase IBs. Their findings demonstrated that these tags significantly influenced the IB’s size and led to enhanced enzymatic activity. This evidence shows that the specific fusion tag strongly influences, rather than strictly dictates, the structural and functional properties of the resulting IBs.

Huang et al. [96] introduced green fluorescent protein (GFP) as an aggregation-promoting fusion partner for the otherwise soluble acid phosphatase PhoC. The work highlights the critical role of linker flexibility in determining the activity and characteristics of final IBs, with rigid linkers showing increased relative specific activities for fluorescence (54.9%), phosphatase (21.1%), and phosphotransferase (8.28%) compared to those with the flexible linker. These results show that linker engineering can modulate IB structure and functional output, with effects dependent on linker properties and growth conditions. Gil-Garcia et al. [112] utilised the natural coiled-coil ZapB domain as an aggregation tag to form α-helix-based IBs that preserved a native-like state with high functional retention. Reduced cytotoxicity and favourable indicators of biocompatibility were reported, supporting the use of modular, non-amyloid aggregation designs.

The work by Sánchez et al. [113] demonstrates that the hexahistidine (6His) tag significantly influences the structural and functional properties of protein-releasing IBs, enabling controlled protein release and tuneable functionalisation through coordination with divalent cations like Zn^2+^. This approach has been proposed for potential applications in drug delivery and regenerative medicine. Carratalá et al. [114] demonstrated that the fusion of aggregation-prone peptides (APPs) like L6K2 to highly soluble recombinant proteins can direct these proteins into IBs. The study found that while L6K2 was effective in reducing the solubility of iRFP, its effect on GFP was not as significant initially. However, by engineering the L6K2 peptide to include repeats of leucine and lysine residues, its aggregating potential could be amplified, leading to increased aggregation propensity for GFP. The authors proposed a mechanism of stereospecific interactions for the formation of hybrid IBs, where co-expression of two different proteins sharing identical aggregation-prone domains allows the protein with higher aggregation propensity to guide the soluble protein into the insoluble fraction.

While these optimisation strategies provide a solid foundation for designing functional IBs or CatIBs, the development of broadly transferable “toolbox” strategies still relies largely on empirical experimentation.

### 4.3. Applications of Functional IBs

IBs produced through the strategies outlined above have been explored across a range of biotechnological and biomedical applications, reflecting material properties that support their use as protein-based biomaterials. Harnessing IBs for direct therapeutic use remains challenging, particularly regarding their functional heterogeneity, potential cytotoxicity, and immunogenicity [115,116]. Biomedical applications of IBs are less developed and largely preclinical, but have been investigated in contexts including cell proliferation, drug delivery, vaccine adjuvants, tissue regeneration, peptide production, and antimicrobial activity [27,116,117]. Functional IBs have been shown to promote cell adhesion, proliferation, and wound healing in vitro, with applications in tissue engineering demonstrated through patterned IB surfaces and enzyme reservoirs [118,119]. IBs have also been engineered as nanoparticulate delivery systems, demonstrating cellular internalisation into mammalian cells and sustained release of therapeutic proteins, with efficacy reported in cancer models and infectious disease vaccine studies [120,121]. Particulate vaccines based on IBs have elicited robust preclinical cellular immune responses, including Th1 and Th17 polarization, and protective effects in animal models [122]. Controlled release of embedded proteins from IBs has been shown to enable sustained therapeutic effects in preclinical regenerative medicine models. IBs have also been used as production platforms for antimicrobial peptides (AMPs), addressing challenges associated with host toxicity and peptide degradation during heterologous expression [117,123]. Recent studies in human cell lines and animal models indicate that IBs and soluble protein nanoparticles are generally non-toxic and biocompatible in the systems tested, with minimal oxidative stress or genotoxicity [115]. Beyond molecular engineering, host strain selection represents an additional factor influencing the biomedical utility of functional IBs. Because endotoxins present in *E. coli*-produced IBs may trigger undesirable immune responses, endotoxin-free hosts such as ClearColi BL21 (DE3) have been explored. This approach can reduce endotoxin-associated safety concerns, supporting the use of IBs in biomedical applications [124]. Across these biomedical studies, IBs function as particulate protein biomaterials whose biological performance is governed less by catalytic optimisation than by material attributes such as particle heterogeneity, release kinetics, and host-derived impurities, which collectively define both therapeutic potential and translational constraints. De novo fabrication of artificial IBs (ArtIBs) has been proposed as a strategy for generating more clinically oriented IB systems. Unlike natural IBs, ArtIBs are assembled from cell-free components at defined stoichiometries, resulting in more consistent and well-characterised compositions. This design has been conceptually linked to endocrine-like release of secretory amyloid granules and may support prolonged protein drug release. These approaches may enhance reproducibility and regulatory readiness in biomedical applications [125].

Across the literature, β-galactosidase is most frequently used as a model enzyme, followed by lysine decarboxylase, alkaline phosphatase, alcohol dehydrogenase, and proteases, to demonstrate CatIB formation, activity, and stability. Conventional studies of CatIBs involved primarily the validation and optimisation of various aggregation-inducing partners in producing efficient CatIBs [27,29,116]. Subsequent studies incorporating cross-linking, co-localisation, magnetic functionalisation, and flow reactor compatibility have reported improvements in biocatalytic metrics, including activity recovery, thermal stability, reusability, and substrate conversion. To improve handling and mechanical stability during repeated usage, Nahálka et al. [99] introduced cross-linked IBs that integrate physical entrapment with chemical cross-linking. CatIBs of sialic acid aldolase (SAA) were entrapped in alginate beads cross-linked with glutaraldehyde. This robust immobilised CatIB system enabled repetitive batch production of sialic acid over up to 20 cycles without statistically significant activity loss.

Magnetic functionalisation has also been explored to facilitate catalyst separation and reuse in dynamic reaction systems. A low-cost magnetisation approach has been demonstrated using CatIBs of UDP-glucose pyrophosphorylase (GalU) and SAA. The method involved mixing CatIBs with magnetic iron oxide particles followed by freezing and gentle lyophilisation. This procedure yields protein-inorganic composite CatIBs that can be separated magnetically without high-speed centrifugation. Using this strategy, GalU-CatIBs were reused up to 50 times in UDP-glucose synthesis, with conversion increasing from 30% to 50% in 1 h cycles, whereas centrifugation-based recovery led to inactivation after 10 cycles. Similarly, SAA-CatIBs maintained ~50% conversion over 14 cycles (4 h each) with minimal protein leakage (2.6–2.8%), and in scaled-up 10 mL reactions, achieved full conversion after 48 h with sustained performance across 10 cycles [71]. In a related approach, Han et al. engineered magnetic cross-linked CatIBs (rCalB-mclIBs) of Candida antarctica lipase B by incorporating Fe_3_O_4_ nanoparticles during oxidised dextran cross-linking. Under optimised conditions, this protein–nanoparticle composite exhibited 113.9% activity recovery following magnetic cross-linking, retained 85.4% activity after 10 reuse cycles, and achieved 84.4% enantiomeric excess (e.e.p), demonstrating operational robustness and stereoselectivity [126]. In contrast to adsorption-based magnetic functionalisation, this chemically cross-linked strategy integrates magnetic responsiveness directly into the CatIB matrix, enhancing mechanical stability at the expense of strict carrier-free composition.

In multi-enzymatic cascades, enzyme co-localisation within CatIBs provides a genetically programmable, system-dependent approach for co-assembling two enzymes within a single aggregate, enabling coordinated cascade operation in demonstrated systems. Jäger et al. [68] fused a coiled-coil domain as an aggregation-inducing tag to co-express and co-immobilise two enzymes, a benzaldehyde lyase and an alcohol dehydrogenase, in *E. coli*, forming Co-CatIBs. This enabled a two-step cascade for (1R,2R)-1-phenylpropane-1,2-diol (PPD) production, a precursor to the calcium channel blocker diltiazem. The Co-CatIBs achieved final product levels comparable to those obtained with soluble enzymes, although product formation was slower. However, both enzymes exhibited substantially higher stability in Co-CatIBs, retaining approximately 7-fold (PfBAL) and 15-fold (RADH) higher residual activities than their soluble counterparts after four days [68]. In another CatIB-based cascade, Kőszagová et al. [127] assembled a multi-enzyme cascade comprising four CatIBs for the conversion of N-acetylglucosamine into UDP-N-acetyl-D-glucosamine. The cascade included CatIBs from two polyphosphate kinases (PPKs) and N-acetylhexosamine 1-kinase (NahK) and N-acetylglucosamine-1-phosphate uridyltransferase (GlmU). The two-PPK system achieved nearly ten-fold ATP regeneration and complete UTP utilisation without detectable nucleotide degradation, supporting efficient cofactor recycling and reduced downstream processing demands.

The transition of CatIBs from batch biotransformations to continuous-flow operation represents an emerging process direction rather than a demonstrated operational standard. Across biocatalytic applications, CatIB performance reflects a balance between aggregation-driven localisation benefits and material-level limitations, including mechanical fragility, leaching propensity, and system-dependent mass transfer, motivating the recurrent use of cross-linking, magnetic composites, or flow-compatible formats in reported designs. An approach by Ölçücü et al. [70] successfully implemented CatIBs in a packed-bed flow reactor for the asymmetric reduction of chloroacetophenone to the corresponding chiral alcohol. The flow system operated for up to 280 h at 30 °C without significant activity loss and maintained high catalytic efficiency, achieving full conversion and enantiomeric excess values of up to 98%, consistent with CatIB compatibility under continuous processing conditions.

Dong et al. [128] explored the application of ST/SC chemistry, derived from Streptococcus pyogenes fibronectin-binding protein, to induce CatIBs-STC formation in *E. coli*. By fusing SpyTag (13-amino acid peptide) and SpyCatcher (116-amino acid domain) [129] to the N- and C-termini of six proteins with varying oligomeric states, ST/SC chemistry was repurposed from cyclization to genetically encoded aggregation, yielding a carrier-free but covalently cross-linked immobilisation strategy. Mechanistically, aggregation correlated strongly with oligomeric state rather than enzyme identity. Monomeric GFP showed low aggregation, with approximately 12.51% of its activity present in aggregates, whereas ~85.44% of octameric leucine dehydrogenase activity was retained in this form. Direct comparisons between traditional cross-linked enzyme aggregates (CLEAs) and CLEA-CatIBs-STC showed improved reusability, thermal stability, and organic-solvent tolerance for CLEA-CatIBs-STC, supporting the integration of genetically encoded aggregation with cross-linking strategies.

Limitations in mechanical stability and leaching have motivated the development of cross-linking strategies for CatIBs. Rational protein design was applied to improve the stability of β-glucosidase CatIBs. By engineering surface carboxyl groups via site-directed mutagenesis (introducing additional glutamate and aspartate residues), researchers significantly improved the efficiency of cross-linking between CatIBs and polyethylenimine. The resulting cross-linked protein aggregates exhibited uniform morphology and enhanced thermal stability, retaining approximately 80% activity after 60 min at 80 °C and around 50% at 90 °C for 40 min, with more than 75% activity after six reuse cycles [130]. Although this approach introduces covalent reinforcement and departs from a strictly carrier-free format, it demonstrates how surface-chemistry-guided cross-linking can balance mechanical robustness and catalytic performance. Table 4 summarises these application-driven strategies, highlighting how cross-linking, magnetic functionalisation, enzyme co-localisation, and reactor integration can improve CatIB performance in biocatalytic applications.

**Table 4:** CatIBs as efficient biocatalytic systems.

Enzyme	Enzyme Class	Fusion Partner/Engineering Modification	System	Yield/Activity Retention	Reusability/Operational Duration	Stability Improvement	Catalytic Metrics	References
Sialic acid aldolase (SAA)	Lyases	N-terminal CBDclos	Alginate cross-linked	13% of total dry cell weight (IB fraction)	20 cycles (~40% conversion)	Minimal protein leakage	~40% conversion	[99]
UDP-glucose pyrophosphorylase (GalU)	Transferases	N-terminal CBDclos	Magnetisation	24.83% specific activity retained ^†^	10 cycles (24 h); 50 cycles (1 h)	No detectable protein release	~60% conversion (24 h); 30–50% (1 h)	[71]
SAA	Lyases	N-terminal CBDclos	Magnetisation	26.68% specific activity retained ^†^	14 cycles (~50% conversion)	~2.6–2.8% protein release over 14 cycles	~45% conversion (1 h); ~50% (4 h)	[71]
Benzaldehyde lyase (PfBAL, *Pseudomonas fluorescens*) and Alcohol dehydrogenase (RADH, *Ralstonia* sp.)	Lyases and oxidoreductases	Tetramerisation domain of tetrabrachion (TDoT) of *Staphylothermus marinus*	Co-localised CatIBs packaging	3.3% (PfBAL)and 1.4% (RADH) residual activity calculated based on *k*_cat_ relative to the corresponding soluble enzyme	ND *	Co-CatIBs showed 7-fold (PfBAL) and 15-fold (RADH) higher residual activity than soluble form after 4 days of reaction	PPD production: ~60 mM (~47% theoretical yield) after 4 days	[68]
Lipase B from *Candida antarctica* (CalB)	Hydrolases	Poly-lysine	Dialdehyde cross-linking and magnetisation	105.2–352.3 mg IBs/0.4 g wet cells; 113.9% activity recovery (optimised)	10 cycles (85.4% relative to initial activity) at 25 °C	86.4% activity retained after 10 days at 4 °C	15.1% conversion; 86.5% ee **	[126]
RADH	Oxidoreductase	N-terminal GFIL8	Silica-assisted packed-bed flow	18% residual activity relative to soluble RADH	Continuous: 280 h (~78% conversion at end)	96% after 5 days at RT	92% yield (120 h), 98% ee; STY ***: 3.55 g·L^−1^·h^−1^	[70]
Leucine dehydrogenase from *Bacillus cereus*	Oxidoreductases	SpyTag/SpyCatcher (ST/SC)	Intermolecular cross-linking	85.44% of crude-extract activity in the aggregated fraction	10 cycles (98.70% relative to initial activity) at 30 °C	Reduced thermal stability vs soluble LDH; improved ethanol/isopropanol tolerance	ND	[128]
β-glucosidase (TsBgl1) from *Thermotoga* sp. KOL6	Hydrolases	Site-directed mutagenesis	Protein engineering and EDC-NHS/PEI cross-linking.	>90% of crude-extract activity in IB fraction	6 cycles (77% initial activity, 80 °C)	4.1-fold higher residual activity after 40 min at 90 °C (E7D3 vs uncross-linked E7D3 CatIBs)	1.7-fold higher glucose conversion than wild-type CatIBs	[130]
Poly-phosphate kinases (SePPK and PPK3): N-acetylhexosamine-1-kinase (NahK) N-acetylglucosamine-1-phosphate uridyltransferase (GlmU)	Transferases	CBDclos, and LHS1 tags (used on different enzymes in the cascade)	Multienzyme conversion of N-acetyl glucosamine into UDP-N-acetyl-D-glucosamine	ND	ND	ND	~10-fold ATP regeneration; 90–100% UTP conversion; STY: 0.253 g·L^−1^·h^−1^)	[127]

* ND, not determined; ^†^ specific activity relative to unmagnetised IBs; ** ee, enantiomeric excess; *** STY, space–time yield.

### 4.4. Limitations and Challenges in Current IB-Based Technologies

Although many engineering concepts for CatIB development show promise, optimisation efforts remain largely enzyme-specific and lack transferable design principles. Challenges in scaling up production while maintaining consistent IB characteristics persist. Research often focuses on a limited set of proteins or fusion tags, thereby restricting extension to diverse protein classes. This methodological constraint reduces the applicability of findings to broader biotechnological contexts and protein engineering strategies. Many studies either do not explore combinatorial variations extensively or do not integrate computational predictions with experimental validation. The impact of fusion tags on protein folding kinetics and cellular quality control mechanisms remains under-investigated, with potential implication for reproducibility and scalability [131]. Although molecular mechanisms underlying IB assembly and activity retention are increasingly understood, predictive modelling and scalable control of IB formation remain limited. Addressing these gaps will likely require advanced structural techniques (e.g., cryo-EM, solid-state NMR) combined with computational modelling to characterise conformational and structural heterogeneity. In terms of industrial applications, many studies are confined to laboratory-scale or in vitro analyses, with scarce data on long-term stability, scalability, and performance of CatIBs in industrial or biomedical settings. This constrains translational potential and practical deployment of IB-based technologies [29].

## 5. Emerging Self-Assembling Strategies for Biocatalysis

Self-assembling strategies based on genetic fusions, peptide tags, and supramolecular interactions enable the formation of ordered assemblies that preserve enzymatic activity and, in specific engineered systems, facilitate substrate channelling and improved laboratory-scale operability. This section examines recent work on modular self-assembling designs that integrate computational modelling and, in some cases, high-throughput screening to optimise assembly with relevance to industrial constraints.

### 5.1. Towards Rationally Engineered Self-Assembling Domains

Reported biocatalytic studies demonstrate that engineered self-assembling proteins can function as carrier-free immobilisation platforms with improved stability, reusability, and efficiency relative to their soluble counterparts. Further discussion focuses on recent advancements in self-assembling systems that have followed rational engineering principles, including control over multivalency, spatial organisation, and recovery modality. A ST/SC system originally developed for site-specific assembly was extended to form catalytically active magnetic protein aggregates (CatMPAs), in which SpyTag-fused ferritin scaffolds (Citrine-EcftnA H34L/T64I) were conjugated after cell lysis with SpyCatcher-fused alcohol dehydrogenase (RADH). The resulting CatMPAs were catalytically active, showing no measurable activity loss over 5 days at room temperature (101.3% retention), compared with 38.7% for free SpyCatcher-RADH. These CatMPAs, purified magnetically from crude extracts with 9.4–18.4% efficiency relative to washed pellets, represent modular, partly ex vivo-assembled immobilisates for biocatalysis, in which magnetic recoverability is integrated directly into the protein scaffold, without ex vivo iron loading [132]. By advancing genetically encoded magnetisation, this approach reduces reliance on external carriers and enables simplified recovery, while remaining subject to scaffold-imposed constraints on enzyme loading and applicability across different enzyme architectures [133]. The development of CatIBs as a robust biocatalytic platform is often hindered by the time-consuming and labour-intensive optimisation required to identify the optimal combination of aggregation tag and target enzyme. In one study, high-throughput screening using Golden Gate Assembly generated 63 BsGDH-CatIB variants. Automated liquid handling and Bayesian optimisation (Thompson sampling) identified top-performing constructs, including TDoT-PT-BsGDH, with fitted reaction rate constants of 0.15–0.19 min^−1^, and showed that rigid PT linkers outperformed flexible designs. This approach reduced manual workload by 88% and enabled systematic structure–function analysis, facilitating more efficient CatIB optimisation [134]. Another approach employed rational design to identify a refined truncated PagP, a β-barrel outer membrane protein, that promotes IB formation in this system through its high β-sheet content and hydrophobicity [123]. Using AGGRESCAN (a web-based tool predicting aggregation propensity from in vivo amino acid tendencies), seven HSs (HS1-HS7) were identified in PagP, with a C-terminal cluster (HS4-HS7) showing high aggregation potential. A proline-rich region in β-strands was noted, consistent with reduced β-sheet stability. Rational substitutions replaced prolines with residues of high β-sheet propensity (e.g., leucine, isoleucine) and hydrophobicity, leading to enhanced aggregation. Optimised PagP variants increased the accumulation of Magainin II fusion proteins by 44.3–60.5% relative to unmodified PagP. While this rational strategy improves mechanistic understanding and design control, its demonstrated applicability remains restricted to high-level AMP production rather than larger globular enzymes [123].

Beyond ST/SC systems and CatIBs, a range of self-assembling strategies have been applied to enzyme immobilisation. Engineered Tetrapeptide Repeat Affinity Proteins (TRAPs) have been used as scaffolds to assemble multi-enzyme systems (e.g., FDH-AlaDH-ωTA), achieving up to 5-fold higher productivity (5.21 g·g^−1^·h^−1^ for L-alanine) and a 4.25-fold yield improvement for benzylamine synthesis via NADH channelling, with solid-support immobilisation yielding 18% L-alanine after the third cycle, compared with 10% for the non-scaffolded system [135]. In a related rational protein-fusion approach rather than a self-assembled scaffold, rationally designed fusion proteins linked ADH and aldDH using cationic linkers to promote electrostatic substrate channelling, resulting in a turnover rate of 5.5 s^−1^ and 90 ± 20% acetaldehyde to ethanol conversion efficiency, and enabling application in bioelectrocatalysis for acetate reduction [136]. Self-assembling peptide tags like ELK16 fused to arginine deiminase formed aggregates retaining 57.34% activity after 10 cycles, converting 100 g·L^−1^ L-arginine to 92.3 g·L^−1^ L-citrulline with enhanced thermal stability at 55 °C [103]. Amphiphilic 18A peptide fusions with lysine decarboxylase, optimised via PT linkers, increased thermostability 4.1-fold and retained 37% activity after 20 cycles [137]. Enzyme-inorganic hybrid nanoflowers, self-assembled with Cu_3_(PO_4_)_2_, boosted soybean peroxidase (SBP) activity by ~446% relative to free crude SBP. This study describes a mild aqueous method to generate enzyme nanoflowers from crude SBP extracted from soybean hull coats, enabling simultaneous enzyme immobilisation and purification with increased catalytic activity [138]. Unlike protein-only self-assembly, these nanoflowers are protein–inorganic hybrid structures. Recent work on nanoflowers systems has also examined enzyme–inorganic carrier-free co-assembly for high loading and stability in biocatalysis and biosensing. Unlike traditional chemical cross-linker-based methodologies, nanoflower systems operate under mild reaction conditions and avoid added cross-linkers; in some cases, the cationic metal ions forming the inorganic component contribute directly to enhanced catalytic activity [139].

By exploiting non-covalent protein–protein interaction domains (PDZ, GBD, SH3), Hertel et al. fabricated all-enzyme hydrogels (AEHs) for continuous biocatalysis. Genetically fusing these motifs to a dual enzyme cascade, alcohol dehydrogenase (ADH) for substrate conversion and glucose-1-dehydrogenase (GDH) for NADPH regeneration, enabled self-assembly into AEHs, which were produced either in *E. coli* cells (in-cell AEHs) or from purified enzymes as monodisperse foams. While in-cell all-enzyme hydrogels (AEHs) did not show clear performance advantages in continuous-flow reactors, AEH foams generated ex vivo showed higher catalytic performance under the tested conditions. Notably, SH3- and PDZ-linked systems maintained >70% conversion over a period of 30 h under selected drying and flow conditions, with drying enhancing mechanical stability and foam reactors achieving approximately twofold higher space–time yields than monolithic AEH reactors [140].

### 5.2. Reversible and Programmable Protein Assembly as an Emerging Design Opportunity

Classic immobilisation strategies often rely on irreversible covalent binding or random adsorption, which can impair enzyme function and limit reusability [141,142]. Although protein scaffolds and self-assembling systems have improved spatial organisation and catalytic throughput, their dynamic disassembly and reassembly under mild conditions are less frequently addressed in immobilisation-focused studies [140,143]. This section focuses on three related concepts: reversible protein assembly, enzyme immobilisation, and dynamic control of multienzyme systems [144,145]. Reversible protein assembly (distinct from reversible enzyme–support immobilisation) refers to the controlled formation and dissociation of protein complexes in response to environmental stimuli, enabling tuneable enzyme spatial arrangement [146,147]. Dynamic control encompasses strategies to modulate enzyme assembly states temporally and spatially, thereby enabling optimisation of catalytic performance [143]. The interplay among these concepts informs the design of biocatalysts with controllable assembly behaviour. A light-inducible, reversible self-assembly system was demonstrated using a dodecamer nitrilase, a multimeric enzyme used in nitrile hydrolysis, leveraging the improved light-inducible dimer (iLID) module. Specifically, nitrilase was genetically fused to the AsLOV2-SsrA domain, a photosensitive component of the iLID system. SspB, a 13 kDa adaptor protein, binds SsrA in a light-dependent manner. Upon 470 nm blue light irradiation, the AsLOV2 domain undergoes conformational changes and exposes the SsrA binding site for SspB, triggering rapid dimerisation and supramolecular assembly into circular, sheet-like structures. Disassembly occurs in the dark, restoring lower-order oligomeric states. The system works both in vitro (cell-free) and in vivo (*E. coli*), maintaining approximately 90% of the initial activity post-assembly, with reversible assembly/disassembly over at least five light/dark cycles and approximately 90% activity retained after four reuse cycles in vitro. Mechanistically, light regulates oligomerisation kinetics through reversible, non-covalent interactions without forming interprotein covalent bonds or external scaffolds. This behaviour enables reversible, light-controlled assembly and disassembly of the enzyme system, with high activity retention and reusability demonstrated across repeated light/dark cycles [147].

Multiple molecular interactions, including irreversible ST/SC covalent bonding, and potentially reversible metal coordination, oligomerisation, phase separation, and light-/ligand-responsive binding modules, have been used to achieve protein assembly for enzyme immobilisation [143,145,147,148]. The Scaffold-Mediated Assembly Regulation Tool (SMART) platform developed by Hu and colleagues [143] employs cell surface display on *Corynebacterium glutamicum* as a scaffold to enable tuneable multi-enzyme co-display through integration of orthogonal cross-linked scaffolds with ST/SC and SnoopCatcher/SnoopTag. Using a two-enzyme (maltooligosyltrehalose synthase, AstreY, and maltooligosyltrehalose trehalohydrolase, AstreZ) sequential cascade, conversion of maltodextrin to trehalose was demonstrated. The engineered enzyme-loaded chimeric scaffold with a SpyCatcher:SnoopCatcher ratio of 1:1 (scaf-I) achieved 20.4 g·L^−1^·h^−1^ productivity, compared with 16.7 g·L^−1^·h^−1^ for the free enzymes, while scaffolds with altered enzyme-loading ratios yielded higher productivities, with scaf-II (2:1) and scaf-III (3:1) exhibiting up to a 67.8% increase relative to scaf-I. When scaled-up in a 5 L bioreactor, the optimised SMART system yielded 215.4 g·L^−1^ trehalose with 71.8% conversion and 14.4 g·L^−1^·h^−1^ productivity. Accordingly, the SMART system provides tuneable stoichiometry for enzyme co-display, which directly influences product titre in multi-enzyme cascades. Although Catcher/Tag bonds are irreversible, SMART enables programmable enzyme stoichiometry and, through a separate Bxb1 recombinase-based genetic circuit, temporal control of enzyme and scaffold expression.

Recently, Yu et al. [148] engineered a glutathione peroxidase mimic by incorporating selenocysteine (Sec) into stabilising protein 1 (SP1) as a scaffold. SP1 forms a dodecameric ring with surface histidines, and addition of Ni^2+^ drives assembly of SP1 rings into nanotubes up to approximately 300 nm long via multivalent metal coordination, restricting substrate access to the Sec active sites. This Ni^2+^-induced assembly creates a reversible spatial barrier that inhibits enzymatic activity (“switch-off”). To restore activity (“switch-on”), EDTA (ethylenediaminetetraacetic acid) is introduced, chelating Ni^2+^ ions and inducing dissociation of the protein nano-assemblies, thereby restoring catalytic activity. The Ni^2+^/EDTA-regulated protein nano-assembly system operates through reversible, non-covalent coordination interactions, enabling repeated activity switching without covalent modification. This spatial-barrier strategy has been proposed as adaptable to other scaffolded enzyme systems in which metal-ion coordination is used to regulate substrate accessibility and enzymatic function.

### 5.3. Outlook

Although protein-based systems such as encapsulins, VLPs, ST/SC systems, amyloid fibrils, and CatIBs have validated protein self-assembly as a viable immobilisation strategy, translation from proof-of-concept to robust and scalable industrial platforms remains unresolved. Progress in this field will depend less on identifying new self-assembling proteins than on establishing predictive and transferable design strategies across enzyme classes. To bridge this gap, future work will need to move beyond empirical trial-and-error toward computationally guided modular design and predictive frameworks.

Recent experimental studies have demonstrated that bacterial microcompartment proteins, such as PduA and PduJ, can form diverse supramolecular morphologies, with single amino acid substitutions altering assembly size and architecture in cell-free protein synthesis systems [149]. By integrating machine learning (ML) algorithms with structural biology data, similar predictive approaches could be extended to assess compatibility between enzymes and self-assembling protein scaffolds, including preferred surface charge or confinement properties. Such a “closed-loop” design framework, in which computational predictions are iteratively validated by high-throughput screening, can support the development of more generalisable immobilisation platforms with reduced enzyme leaching and improved activity retention [150]. The integration of rational protein design, data-driven modelling, and synthetic biology offers a route towards more controllable and application-specific self-assembly platforms for enzyme immobilisation in industrial contexts.

Recent work has demonstrated a high-throughput iterative pipeline combining artificial intelligence (AI)-guided de novo design with all-atom molecular dynamics (MD) simulations to systematically elongate β-strands and maximise hydrogen bonding [151]. Although this strategy was not developed in the context of CatIBs, maximising backbone hydrogen-bond networks in force-bearing β-strands constitutes a stabilisation principle that could be investigated for engineering more robust protein components of self-assembling biocatalysts. In Zheng et al., this approach enabled the design of ultra-stable proteins with exceptional mechanical and thermal resistance, retaining their structural and mechanical properties after exposure to 150 °C for 1 h. Selected designed proteins were also incorporated into SpyTag/SpyCatcher-linked hydrogels, which retained structural integrity after heating to 121 °C, demonstrating their potential as building blocks for thermally stable protein assemblies [151].

Translating these design principles to CatIB-forming enzymes remains unexplored, but such strategies could inform the engineering of aggregation-prone enzymes that retain activity under extreme conditions. The reported design–build–test workflow, integrating RFdiffusion for backbone generation, ProteinMPNN for sequence optimisation, and AlphaFold2 for structural validation, outlines a computational pipeline that may be adapted for enzyme systems intended for immobilisation or aggregation. In this context, combining designed enzyme cores with CatIB-inducing tags (e.g., cellulose-binding domains [94], coiled-coil domains such as ZapB [112], or short aggregation-prone peptides [70,111] could be explored to ensure that enhanced stability does not compromise aggregation behaviour. While these approaches are expected to accelerate the rational design of self-assembling biocatalysts, their application to CatIB systems has yet to be experimentally demonstrated. Future investigations are likely to integrate such computational pipelines with scalable experimental platforms, including cell-free protein synthesis (CFPS) systems, which have enabled rapid production and evaluation of genetically engineered self-assembling protein variants in vitro [149]. By enabling high-throughput screening under controlled conditions, CFPS-based workflows can support the experimental evaluation of predictive design principles in industrially relevant biocatalytic systems.

## 6. Conclusions

This review positions protein self-assembly as a unifying design strategy in biocatalysis to generate robust and efficient enzyme immobilisation systems across diverse platforms. Emerging approaches, including synthetic protein nano-compartments, ST/SC systems, and bio-orthogonal phase-separation strategies, enable the organisation of multi-enzyme cascades with programmable spatial arrangement and controllable interactions, reflecting selected organisational features of natural metabolic compartmentalisation. Despite these advances, achieving precise control over enzyme stoichiometry, balancing scaffold robustness with functional flexibility, and ensuring adequate substrate accessibility within assembled systems continue to represent important challenges for future investigation. These challenges are influenced by assembly morphology, porosity, heterogeneity, and associated mass-transfer constraints.

From a process perspective, self-assembling enzyme systems such as CatIBs offer practical advantages including simplified recovery, improved operational stability, and potential for repeated use without additional carrier materials. These features may reduce downstream processing requirements and associated costs compared to conventional immobilisation strategies. However, their performance remains strongly dependent on protein architecture and system-specific constraints, and further work is required to evaluate robustness under industrially relevant operating conditions.

CatIBs catalytic performance depends critically on fusion design parameters, including linker composition, aggregation-inducing tags, and expression architecture, necessitating empirical optimisation and systematic screening. Modular design strategies based on coiled-coil domains, leucine zippers, and amyloidogenic sequences enable more controllable and system-specific assembly. However, a deeper understanding of how structural stability, enzyme accessibility, and local packing density collectively influence enzymatic functionality under immobilised or aggregated conditions is still required.

Recent advances in high-throughput screening, automation, and Bayesian optimisation have accelerated the identification of effective fusion constructs and assembly configurations. Nevertheless, translating these insights into broadly predictive and transferable design frameworks remains an open challenge, as many current solutions remain enzyme- and context-specific. The literature reviewed here indicates clear progress in applying biological self-assembly to biocatalysis, particularly for enzyme immobilisation and multi-step catalytic processes. Further progress will depend on improved mechanistic understanding, predictive design capability, and scalable implementation for biocatalytic platforms relevant to industrial biotechnology and synthetic biology.

## Data Availability

Data sharing is not applicable to this article as no new data were created or analysed in this study.
